# Estimation of Survival Probabilities for Use in Cost-effectiveness Analyses: A Comparison of a Multi-state Modeling Survival Analysis Approach with Partitioned Survival and Markov Decision-Analytic Modeling

**DOI:** 10.1177/0272989X16670617

**Published:** 2016-10-04

**Authors:** Claire Williams, James D. Lewsey, Daniel F. Mackay, Andrew H. Briggs

**Affiliations:** Health Economics and Health Technology Assessment, Institute of Health and Wellbeing, University of Glasgow, Glasgow (CW, JDL, AHB); Public Health, Institute of Health and Wellbeing, University of Glasgow, Glasgow (DFM)

**Keywords:** oncology, survival analysis, Markov models, cost-effectiveness analysis

## Abstract

Modeling of clinical-effectiveness in a cost-effectiveness analysis typically involves some form of partitioned survival or Markov decision-analytic modeling. The health states progression-free, progression and death and the transitions between them are frequently of interest. With partitioned survival, progression is not modeled directly as a state; instead, time in that state is derived from the difference in area between the overall survival and the progression-free survival curves. With Markov decision-analytic modeling, a priori assumptions are often made with regard to the transitions rather than using the individual patient data directly to model them. This article compares a multi-state modeling survival regression approach to these two common methods. As a case study, we use a trial comparing rituximab in combination with fludarabine and cyclophosphamide v. fludarabine and cyclophosphamide alone for the first-line treatment of chronic lymphocytic leukemia. We calculated mean Life Years and QALYs that involved extrapolation of survival outcomes in the trial. We adapted an existing multi-state modeling approach to incorporate parametric distributions for transition hazards, to allow extrapolation. The comparison showed that, due to the different assumptions used in the different approaches, a discrepancy in results was evident. The partitioned survival and Markov decision-analytic modeling deemed the treatment cost-effective with ICERs of just over £16,000 and £13,000, respectively. However, the results with the multi-state modeling were less conclusive, with an ICER of just over £29,000. This work has illustrated that it is imperative to check whether assumptions are realistic, as different model choices can influence clinical and cost-effectiveness results.

Partitioned survival^1-3^ and Markov decision-analytic modeling^4-7^ are two methods widely used in cost-effectiveness analysis. In oncology, the three health states—progression-free, progression and death—are frequently of interest. Partitioned survival only considers the two curves for progression-free survival and overall survival directly, with time in progression calculated using the difference in area between the two other curves. In contrast, Markov decision-analytic modeling studies the clinical pathway of disease by considering the three states of progression-free, progression and death and the relevant transitions between them. All three relevant transitions are considered simultaneously rather than the separate modeling of the two outcomes in partitioned survival. Markov decision-analytic models are typically built in a spreadsheet-based package over discrete time cycles using cohort simulation, and *a priori* assumptions are made with regards to the transition probabilities. Such assumptions are based on what the modeler deems appropriate, and therefore may not be based directly on the observed data for every transition. For example, background mortality rates are sometimes used to inform transition probabilities. In this article, we use the alternative approach of multi-state modeling for comparison with partitioned survival and Markov decision-analytic modeling. Like Markov decision-analytic modeling, multi-state modeling is a state-transition modeling approach and, as such, models each of the transitions of interest simultaneously. However, it uses a continuous-time framework. Typically, the individual patient-level data is used to build survival regression models for each of the transitions and therefore the modelling is based wholly on the observed data. There has been increased awareness of multi-state modeling in the health economics literature;^8-10^ however, the method is still not commonly applied. In one study,^8^ the authors described the use of tunnel states in Markov decision-analytic modeling as a way of building semi-Markov models that relax the Markov property. They implemented a semi-Markov approach that represented tunnel states in an alternative way by using multi-dimensional transition matrices. In another study,^9^ the authors used a multi-state model to inform a microsimulation model for cost-effectiveness analysis. It used exact times of transitions and, as such, negated discrete cycles and provided an alternative to tunnel states, thereby simplifying the process in situations with many health states. The mstate ^11^ package in R ^12^ was used for their multi-state modeling, and Excel for their microsimulation model. In this paper, we build on previous work to calculate transition probabilities. We adapt the existing functionality of mstate based on semi-parametric Cox regression to incorporate parametric regression of transition hazards, with a range of standard distributions, to allow for extrapolation of survival outcomes and hazards that vary over time. Our illustration of the multi-state modeling approach involves this extrapolation and calculates mean Life Years/QALYs in relevant health states for use in cost-effectiveness analysis. Our approach focuses entirely on multi-state modeling and therefore provides an alternative to microsimulation. Its implementation in R is described elsewhere,^10^ allowing other modelers to adopt the approach.

Extrapolation of survival has received much attention in the health economics literature in recent years.^13-18^ Extrapolation of survival is often needed because observations in clinical trials are frequently not of sufficient length to follow each patient to end of life. Latimer^13^ and Bagust^14^ have debated approaches to estimating survival from Kaplan-Meier curves for outcomes used in partitioned survival, such as overall survival and progression-free survival. One way of extrapolating survival is to use parametric regression. The multi-state modeling approach presented in this article extends this to state-transition modeling. The contribution of this paper is to compare this multi-state modeling approach to the two other common methods of partitioned survival and Markov decision-analytic modeling, with a particular emphasis on the different assumptions used with each of the approaches.

## Data and Methods

### Dataset Used for Illustration

To compare the multi-state modeling framework with the two common approaches of partitioned survival and Markov decision-analytic modeling, a specific National Institute for Health and Clinical Excellence (NICE) technology appraisal is used for illustration - TA174.^19^ The economic model submitted by the manufacturer Roche to evaluate the cost-effectiveness of rituximab for the first-line treatment of chronic lymphocytic leukemia is used as an example.^20^ Specifically, the three-state Markov decision-analytic model developed by Roche is compared to partitioned survival and multi-state modeling.

The main source of data in this Markov decision-analytic model is the CLL-8 trial.^21^ It compared rituximab in combination with fludarabine and cyclophosphamide (RFC) v. fludarabine and cyclophosphamide alone (FC) for the first-line treatment of chronic lymphocytic leukemia. The trial had the outcomes progression-free survival and overall survival for each patient, allowing focus to be on the three states of progression-free, progression and death (and the transitions between them). There were 408 patients in the RFC arm and 409 patients in the FC arm. There were 106 progressions, 23 deaths after progression and 21 deaths without progression amongst those in the RFC arm. In the FC arm, there were 148 progressions, 27 deaths after progression and 26 deaths without progression.

Patients were in the trial for up to 4 years and not all of them were observed to the end of their life. It was estimated that only 1.3% of the cohort would survive beyond 15 years [20: p109] and this informed the manufacturer’s decision to use a 15-year time horizon. We used data from the same trial for the partitioned survival and multi-state modeling approaches and used the same time horizon as the manufacturer for comparison purposes.

### Estimation of Mean Survival

Estimates of mean survival were obtained by calculating the area under the extrapolated survival curves. All areas under the survival curves were calculated using the trapezoidal rule with increments of 1/12 years, equivalent to the cycle length of a month in the manufacturer’s Markov decision-analytic model. However, due to computational issues with the Gompertz distribution, the calculation of transition probabilities with the multi-state modeling used increments of 1/12 years up to 9 years followed by increments of 1/144 years up to the 15-year time horizon. This shortening of the cycle length after 9 years was needed to overcome a difficulty in meeting the requirement that differences in cumulative hazards between consecutive time points were below one. After this adjustment to allow calculation of transition probabilities, only the probabilities at 1/12-year increments were involved in the trapezoidal rule calculations, consistent with other approaches. Our choice of 1/144-year increments was based on the ease of calculation of multiples of 1/12. For each of the three approaches, results for mean Life Years and QALYs are presented for each treatment arm. All Life Year and QALY calculations were discounted at an annual rate of 3.5%, the approach taken by the manufacturers in their Markov decision-analytic model.

The next three sections detail each of the approaches in turn.

#### Partitioned Survival

Partitioned survival involves partitioning overall survival into states of interest. As the three states of interest in this illustration were progression-free, progression, and death, the required partitioning of overall survival is achieved using progression-free survival. The two trial outcomes progression-free survival and overall survival were each modeled directly using parametric regression to allow for extrapolation. This approach did not consider post-progression survival directly. Instead, the mean time in progression was derived from the difference in the area under the two survival outcomes that were considered directly. We considered six standard distributions in the modeling for each trial outcome: exponential, Weibull, Gompertz, log-logistic, log normal and generalized gamma. The assessment of fit to the observed data for each of the predicted probabilities was based on AICs, Cox-Snell residuals and visual comparison with Kaplan-Meier estimates.

Initially, for the two survival outcomes considered directly, we carried out extrapolation by fitting parametric regressions to the whole Kaplan-Meier curve and extending the predictions out to 15 years. For progression-free survival, this approach produced predictions that adequately represented survival of zero upon extrapolation to 15 years. However, this was not the case for overall survival. There was a high level of censoring for overall survival. By the end of the trial observation period of 4 years, there were still 85% of patients alive. None of the extrapolations using any of the distributions produced survival probabilities close to zero by 15 years, with 50% being the lowest achieved. Therefore, for overall survival, extrapolation based on starting from the tail end of the Kaplan-Meier curve was undertaken. An approach, outlined by Tappenden and others,^16^ was used to fit a linear regression to the tail of the Kaplan-Meier curve and then back-transform the predictions to the equivalent using a parametric regression. This approach accommodated fitting regressions when the starting time point was beyond zero and survival had dropped below one, as is the case in the tail of a Kaplan-Meier curve. It involved rearranging the survival function of the parametric distribution into a function that had a linear, or other simple relationship, with time. For example, when an exponential fit to the tail data was desired, the exponential survival function S(t) = e^-λt^ was rearranged into log(S) = –λt, which meant the linear regression log(S) ~ t could be performed, with the intercept constrained to be zero. The coefficient for t in this linear regression was then used as –λ in the exponential survival function. Similarly, a rearrangement of the Weibull survival function S(t) = $e^{-\lambda t^{\gamma }}$ meant the line log(-log(S)) = a + b log(t) could be fitted using linear regression, with the coefficients of a and b representing log λ and γ, respectively. Time t was measured from the start of the tail. The regression that was fitted to the tail was then extrapolated to 15 years. Each of the treatment groups was considered separately. To decide the eventual “tail end” of the Kaplan-Meier curve to use, each of the unique observed times was considered as a starting point for the tail. The starting point chosen was the latest time that resulted in an extrapolation that reached zero by 15 years. This starting point produced predictions that adequately represented a time horizon of 15 years for the FC arm but not the RFC arm. Therefore, for the RFC arm, the survival probabilities in the extrapolated period were derived by multiplying the logarithm of the extrapolated probabilities in the FC arm by the treatment hazard ratio from the observed period, and then taking the exponential of the result.

#### Markov Decision-Analytic Modeling Approach Adopted by the Manufacturer

**Figure 1:**
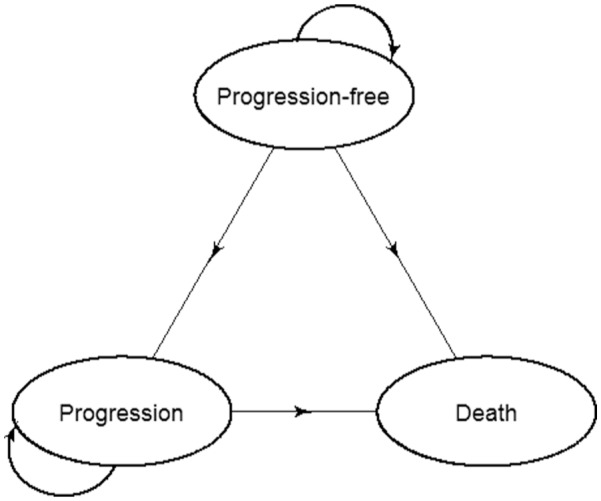
Markov decision-analytic model diagram.

The three-state Markov decision-analytic model used by the manufacturer in their economic evaluation submitted to NICE is shown in Figure 1.

The three transitions—progression-free to progression, progression-free to death without progression, and progression to death—were modeled. The model measured time in discrete monthly cycles. The manufacturers took the usual approach in Markov decision-analytic modeling of assigning transition probabilities before modeling started. The assumptions made by the manufacturer for each of the transitions were as follows:

- Progression to death

A monthly probability of 0.0405 was used, the same for each arm. It was based on an assumption of a constant death rate that was derived from the inverse of the mean of 24.1791 months from the Kaplan-Meier estimate of post progression survival.

- Progression-free to death

This was the observed rate of death while progression-free, or an age-specific background mortality rate,^22^ whichever was largest. The observed monthly probability of death whilst progression-free was 0.0012 and 0.00139 in the RFC and FC arms, respectively.

- Progression-free to progression

This was calculated by adding together the probability for progression-free to death and the probability of staying in the progression-free state, and then subtracting the result from one. The probability of staying in the progression-free state was based on a Weibull regression fitted to the observed progression-free survival data that was then extrapolated to 15 years. This was identical to the Weibull regression used for progression-free survival in the partitioned survival approach.

The model was built in Excel following a cohort of patients from the initial progression-free state over a series of cycles, with movement between states based on the transition probabilities already assigned. Extrapolation was performed by extending the probabilities to the target time horizon of 15 years. The manufacturer used a “clock-reset” approach in that, for the modeling of the progression to death transition, time was set back to zero as patients first entered the progression state. In the continuous time state-transition modeling framework, resetting the clock in this way is considered a semi-Markov approach. However, the approach used was not semi-Markov in the typical sense under the discrete time framework. This was because it did not involve tunnel states or the use of multi-dimensional transition matrices to incorporate time dependency, and only involved simulation of a single cohort.

#### Multi-state Modeling

The multi-state modeling approach fits the same model as the Markov decision-analytic modeling above, in that it uses the same three states and transitions between them. Unlike with the approach commonly used in Markov decision-analytic modeling, this multi-state modeling approach builds survival regression models of each of the transitions directly using the individual patient data. It uses the exact time of transitions and therefore is a continuous time state-transition framework, rather than the discrete time framework used in Markov decision-analytic modeling. The background to the method is explained in a tutorial by Putter and others,^23^ on which much of the explanation in this section is based.

As with Markov decision-analytic modeling, two types of models can be fitted with this multi-state modeling approach: Markov and semi-Markov models. Further details are available elsewhere.^23^ With each of these models, it is possible to build state-arrival extended models. The term “state-arrival extended (semi-) Markov” is described in the tutorial by Putter and others^23^ as a model of an:

“i → j transition hazard that depends on the time of arrival at state i.”

It involves including in a model a covariate that represents patients’ histories and, as such, provides a useful tool to help decide whether the Markov property holds. The effect size and statistical significance of the covariate (which could be time in the previous state, or any function thereof) can aid the decision.

As a preliminary analysis, a Cox state-arrival extended model for progression to death— including a covariate for the time in the previous state—was fitted. This was purely for the purpose of aiding the decision of whether to accept the Markov assumption. It was a Cox model in the sense that the baseline hazard did not follow a specified distribution. It was strictly a Markov, rather than semi-Markov, model because time was measured from first entering the initial (progression-free) state. It was only this transition that affected the decision of whether the Markov assumption was reasonable, as the other transitions did not involve any history as they started in the initial state.

We found evidence to suggest the Markov property did not hold (not shown) and proceeded to use a semi-Markov approach. For each of the transitions, a parametric approach to regression was then taken to allow extrapolation of survival. In a similar manner to the manufacturer’s Markov decision-analytic model, extrapolation was carried out by extending the underlying distribution used for the hazard to the target time horizon of 15 years. Standard distributions were considered for the modeling of each transition: exponential, Weibull, Gompertz, log-logistic, log normal and generalized gamma. AICs are often used to decide between different models. However, they only provide information about the fit to the observed data, and not on how reasonable the extrapolation looks. In addition, we did not consider AICs, or other similar criteria, because the approach involved modeling transition hazards in a competing risks scenario. When competing risks are involved, there is no longer the one-to-one relationship between the hazard and survival probabilities that there is in the absence of competing risks. That is to say, the hazard of a particular event cannot simply be derived from the probability of the survival, because survival is based on a combination of two or more hazards rather than just one. With competing risks, the term survival is reserved for survival free from any of the events (event of interest or competing). The concept of cumulative survival from a particular event is not meaningful in a competing risks setting, as it does not recognize that competing events can also occur. Instead, the cumulative incidence of a particular event is used, as it recognizes that other events can occur, through the use of survival free from any of the events in its calculation. In addition, the effect of a covariate on a hazard of an event can be different from its effect on the cumulative incidence of the event, due to the effect of the covariate on the hazard of a competing event. More generally, in multi-state models, state occupancy probabilities involve combining the hazards for each transition into that state. Therefore, comparing AICs of models for hazards of individual transitions does not correspond to assessing the state occupancy probabilities that are ultimately of interest. AICs can be used in competing risks scenarios when cumulative incidences are modeled directly, such as in the Fine and Gray subdistribution hazards model.^24^ However, there is no known equivalent to AIC, or other similar criteria, appropriate for this approach. Instead, the resultant model was chosen based on a visual assessment of relevant plots that achieved a balance of a good fit to the observed data and a reasonable extrapolation to 15 years.

### Software Used

All analysis was carried out using R version 3.0.1.^12^ The multi-state modeling was undertaken using the mstate package.^11^ Adaptations were made by the authors to accommodate parametric regression rather than the Cox semi-parametric approach. The phreg and aftreg functions in the eha package^25^ and the flexsurvreg function in the flexsurv package^26^ were used to fit parametric regression models. The phreg function was used to fit exponential, Weibull and Gompertz proportional hazards models. Accelerated failure time log-logistic and log-normal models were fitted using the aftreg function. The flexsurvreg function was used to fit generalized gamma models. The Cox cumulative hazards were replaced with parametric equivalents and were used as arguments in the mssample function for prediction purposes. 5000 simulations were used with the mssample function to sample paths from the multi-state model. The mssample function simulates all relevant paths (all possible transition journeys) through the multi-state model in order to calculate transition/state occupancy probabilities.^27^ Areas under the extrapolated survival curves were estimated using the trapz function in the caTools package.^28^ A tutorial explaining how to implement the approach is detailed elsewhere.^10^

## Results

### Modeling Results for Each Approach

Tables 1A and 1B show the results of modeling from the partitioned survival and multi-state modeling approaches, respectively. For the modeling of progression-free survival in the partitioned survival approach, a single Weibull regression was used which was extrapolated to 15 years. For the modeling of overall survival in the partitioned survival approach, the observed and extrapolated sections were based on different models. The observed section was based on a single exponential model. A Weibull model was used for the extrapolated section of FC and then the treatment hazard ratio in the observed period was applied to the FC probabilities to obtain those for RFC.

**Table 1A:** Partitioned Survival Modeling Results

**Partitioned Survival**	Distribution	Coefficient	SE	HR (95%) CI	*P* Value
*Progression-free survival* *(event: progression or death)*	*Weibull*				
Treatment: RFC v. FC		−0.519	0.117	0.595 (0.473, 0.748)	<0.001
Log(scale)		1.237	0.060		
Log(shape)		0.310	0.051		
*Overall survival* *(event: death with or without progression)*					
Observed period (0–3.6 years)	*Exponential*				
Treatment: RFC v. FC		−0.284	0.204	0.753 (0.505, 1.123)	0.164
Log(scale)		2.753	0.137		
Extrapolation (3.6–15 years)^a^					
Weibull shape		2.257	0.484		
Weibull log(scale)		−4.377	0.659		

a Derived from a linear regression using the approach described elsewhere.^16^

**Table 1B:** Multi-state Modeling Results (Gompertz Distribution Used for Each Transition)

**Multi-state Model**	Coefficient	SE	HR (95%) CI	*P* Value
Progression-free → progression				
Treatment: RFC v. FC	0.542	0.128	0.572 (0.446, 0.735)	<0.001
Shape	0.474	0.068		
Log(scale)	−2.187	0.13		
Progression-free → death without progression				
Treatment: RFC v. FC	−0.343	0.294	0.710 (0.399, 1.262)	0.243
Shape	−0.487	0.207		
Log(scale)	−2.825	0.265		
Progression → death				
Treatment: RFC v. FC	0.342	0.285	1.408 (0.806, 2.461)	0.229
Shape	0.174	0.244		
Log(scale)	−1.627	0.267		

The modeling of progression-free survival (i.e., the composite event outcome progression or death without progression) using the partitioned survival approach resulted in a hazard ratio (95% CI) of 0.595 (0.473, 0.748), indicating a reduced risk for the RFC group. This was very similar to the hazard ratio of 0.572 (0.446, 0.735) from the modeling of progression-free to progression using the multi-state modeling approach. This was because the composite event progression or death without progression was dominated by progression events.

The modeling of overall survival (i.e., the event outcome death with or without progression) using partitioned survival over the observed period of the trial had a hazard ratio of 0.753 (0.505, 1.123) for RFC v. FC. When this outcome was split into the two transitions—progression-free to death without progression and progression to death—using multi-state modeling, a change in the direction of the effect was apparent for progression to death. However, there was no evidence of a statistically significant treatment effect for either of the two transitions involving death in the multi-state modeling approach or the modeling of death using the partitioned survival approach.

The Markov decision-analytic modeling assumed no treatment effect for progression to death—equivalent to a hazard ratio of 1 with no uncertainty whereas the multi-state modeling resulted in a treatment hazard ratio of 1.408 (0.806, 2.461). No other transitions in the Markov decision-analytic modeling could be expressed as the equivalent of a hazard ratio because the transitions were not modeled using regression. The probabilities of transition were instead based on the manufacturer’s assumptions.

### Visual Assessment of the Fits

In this section, the fit from each of the modeling approaches was assessed informally by inspecting relevant plots.

#### Probability of Being in Progression

**Figure 2:**
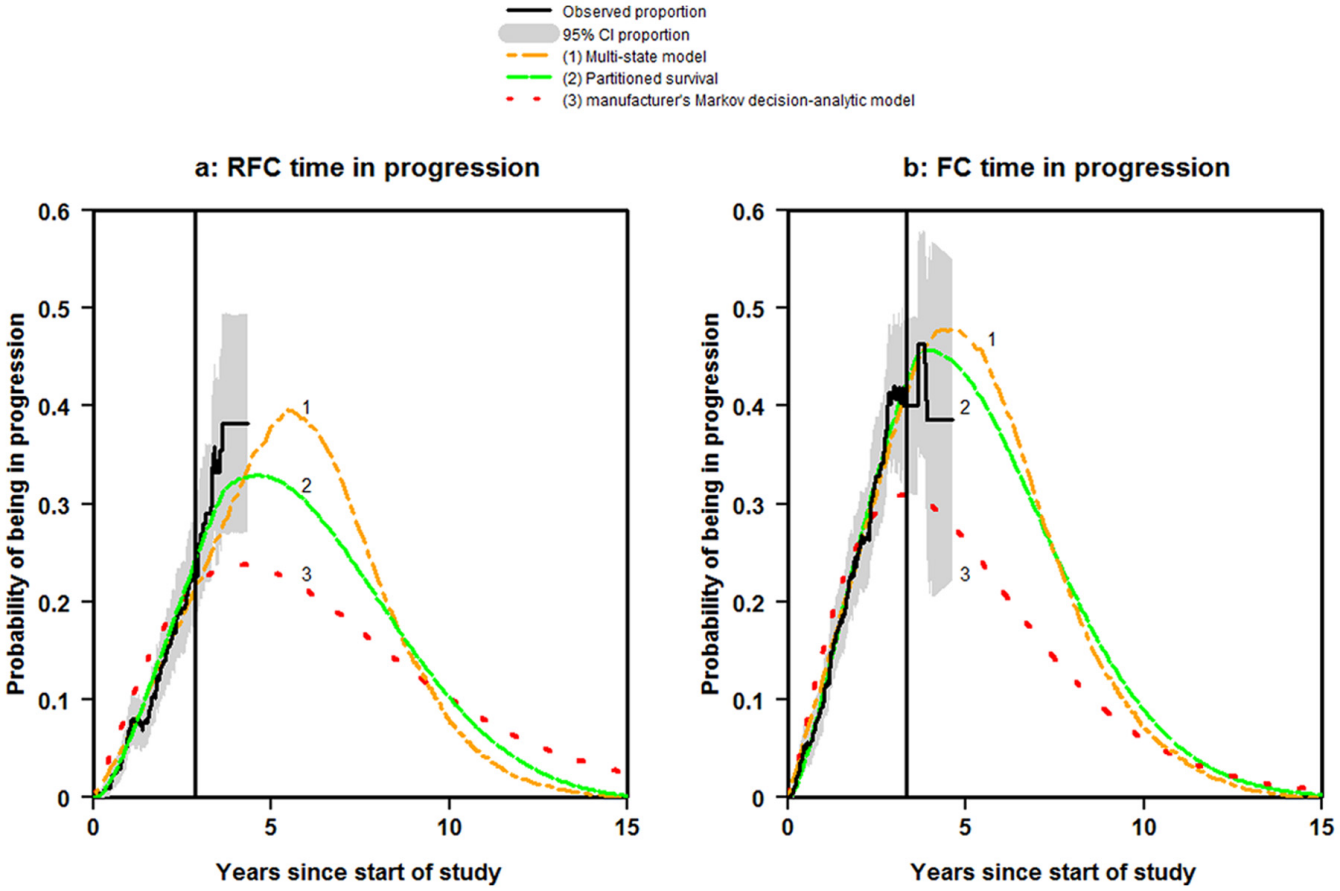
Time in Progression.

The vertical solid lines in Figure 2 (a) and 2 (b) show the times at which there were less than 20 patients at risk of death after progression and therefore the proportion estimates were less reliable. They therefore provide a dividing line between the periods of observation and extrapolation. In addition, the shaded areas show the 95% CI for the observed proportions (created using 5000 bootstrapped samples).

For the RFC arm, all approaches provided a good fit to the observed data up until the vertical line (Figure 2a). After this point, there was a marked difference between the approaches in where and when the predictions of being in progression peaked. The partitioned survival approach and the multi-state modeling were the only methods to reach zero by 15 years.

For the FC arm, the partitioned survival and multi-state modeling approaches provided a good fit to the observed data up until the vertical line (Figure 2b). The Markov decision-analytic modeling only provided a good fit to the observed data up to 2.3 years. Again, there was a marked difference between the approaches in the peaks. All three approaches reached zero by 15 years as required.

#### Progression-free to Death Without Progression

**Figure 3:**
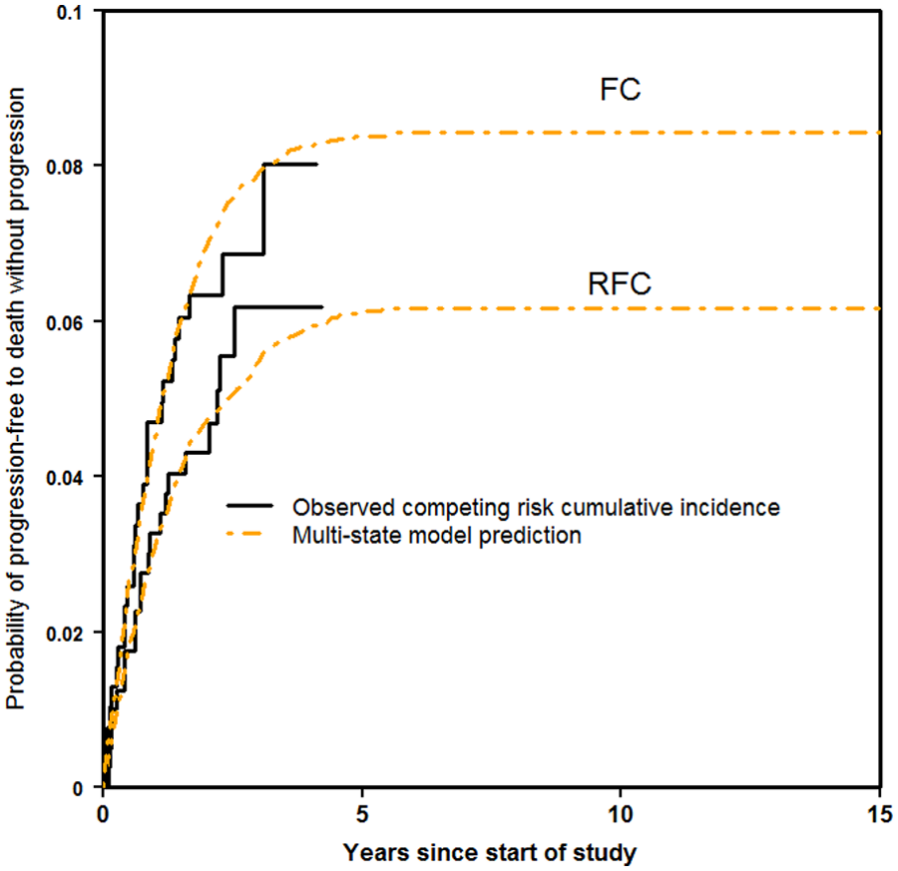
Progression-free to death without progression.

Figure 3 shows, for each treatment arm, the competing risk cumulative incidence estimate of Progression-free to death without progression together with the predictions from the multi-state modeling. It can be seen that the multi-state modeling fitted the observed data moderately well. These predictions were possible because the multi-state modeling allowed the state occupancy probabilities of death to be split into death without progression and death after progression.

#### Progression to Death

**Figure 4:**
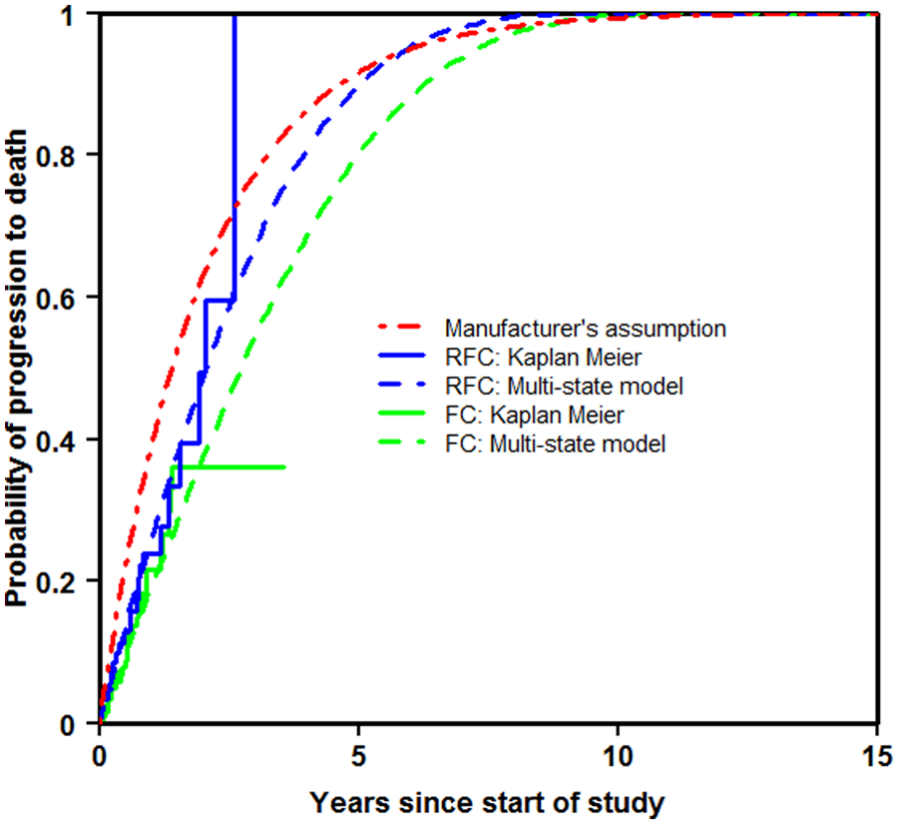
Progression to death.

Figure 4 shows, for each treatment arm, the Kaplan-Meier estimate of progression to death together with the predictions from the multi-state modeling and the manufacturer’s assumption. It can be seen that the extrapolation for both methods was fairly good at reaching one by 15 years. For RFC, the multi-state modeling fitted the Kaplan-Meier estimate quite well, and FC to a lesser extent. However, the manufacturer’s assumption was less well-fitting to each of the treatment arms.

#### Progression-free Survival

**Figure 5:**
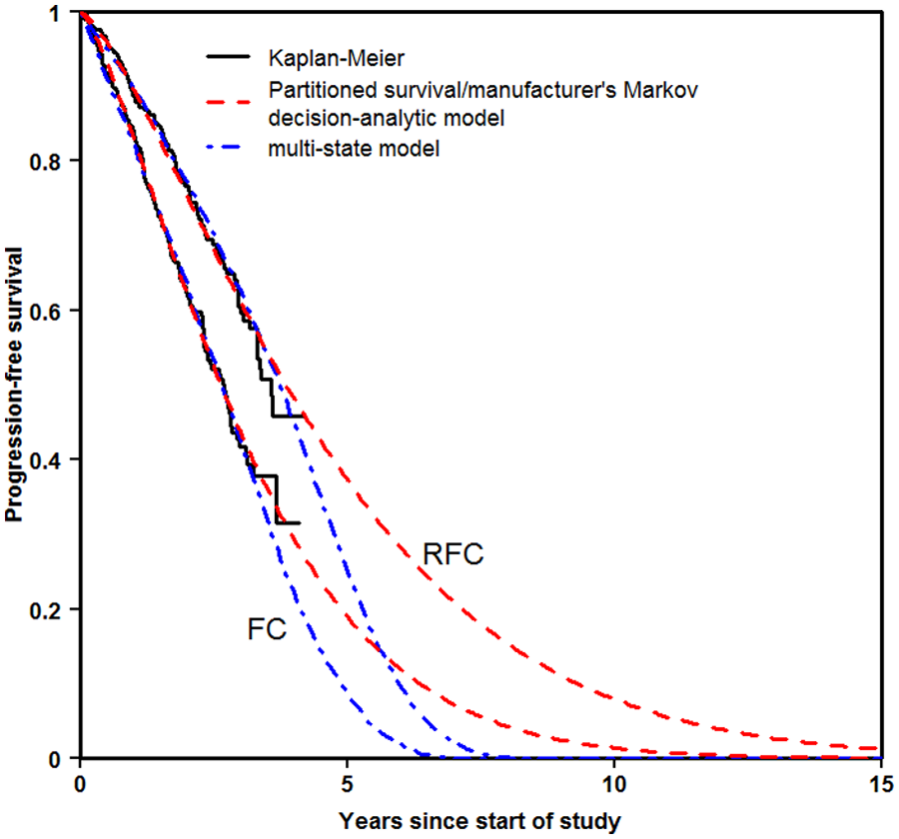
Progression-free survival.

The partitioned survival approach and the Markov decision-analytic modeling were based on the same fit to progression-free survival and therefore only one curve is shown to represent both approaches (Figure 5). For both treatment arms, each of the three approaches appeared to provide a good fit to the Kaplan-Meier estimate. The extrapolation using multi-state modeling reached zero somewhat earlier than the other approaches. This seems more plausible, as it allows those patients who reach progression to spend time in that state before reaching the absorbing state death. It is compatible with all patients reaching death by 15 years.

#### Overall Survival

**Figure 6:**
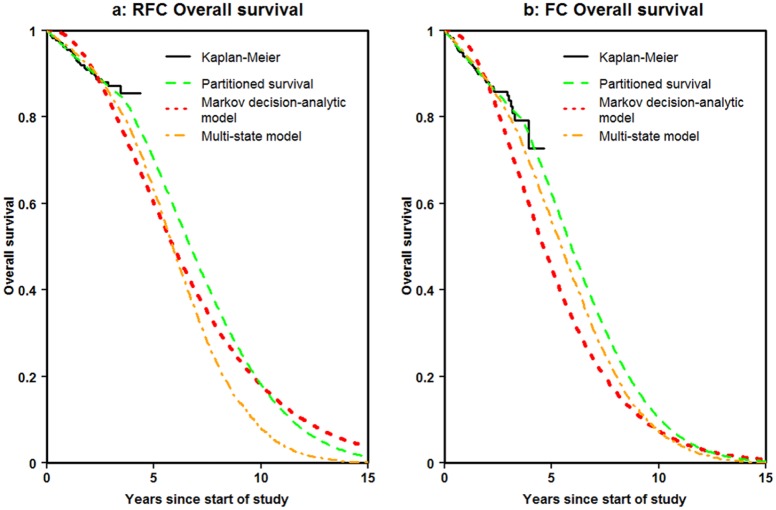
Overall survival.

Figures 6 (a) and 6 (b) show the Kaplan-Meier estimates of overall survival together with the predictions from each of the modeling approaches for the RFC and FC arms, respectively. The partitioned survival and multi-state modeling fitted the observed data over the first 4 years reasonably well. The Markov decision-analytic modeling overestimated survival over the first 2 years. All approaches reached zero by 15 years for FC. However, the multi-state modeling was the only approach to do so for RFC.

### Comparison of Mean Life Years/QALYs

The comparison presented in this article is not a congruous comparison, because the approaches did not make all the same assumptions with regards to the transition probabilities/hazards. However, as can be seen in Appendix 1, a model built using this multi-state modeling approach and one created using the common approach of Markov decision-analytic modeling produced very similar results when they made the same assumptions. We now comment on how the different assumptions influenced the differences in results between the approaches.

Table 2 shows the mean Life Years and QALYs for each of the approaches. Mean QALYs were calculated by assuming a utility of 0.8 for the time spent progression-free and 0.6 for the time spent in progression, the approach used by the manufacturer in the Markov decision-analytic model.

**Table 2:** Mean Life Years and QALYs

	Partitioned Survival			Markov Decision-Analytic Model			Multi-state Modeling		
	RFC	FC	Incremental	RFC	FC	Incremental	RFC	FC	Incremental
**Mean Life Years**	**5.96**	**5.31**	**0.65**	**5.73**	**4.65**	**1.07**	**5.29**	**4.97**	**0.32**
Mean Life Years Progression-free	4.10	2.92	1.18	4.11	2.93	1.18	3.35	2.55	0.81
Mean Life Years in Progression	1.86	2.39	−0.53	1.62	1.73	−0.11	1.93	2.42	−0.49
**Mean QALYs**	**4.40**	**3.77**	**0.63**	**4.26**	**3.38**	**0.88**	**3.84**	**3.49**	**0.35**
Mean QALYs Progression-free	3.28	2.34	0.95	3.29	2.34	0.94	2.68	2.04	0.65
Mean QALYs in Progression	1.11	1.43	−0.32	0.97	1.04	−0.07	1.16	1.45	−0.29

Table 2 shows that the manufacturer’s Markov decision-analytic modeling resulted in larger overall benefits than any of the other approaches. This was primarily due to that approach producing smaller decrements whilst in progression of -0.11 for mean Life Years and -0.07 for mean QALYs. This reflected the manufacturer’s assumption of no treatment effect for the progression to death transition. They based this decision on the log-rank test for the difference in Kaplan-Meier estimates of post-progression survival, which did not provide evidence of a statistically significant effect of treatment (*P* = 0.395). In contrast, the modeling of the progression to death transition using the multi-state modeling resulted in a hazard ratio of 1.408 for treatment. This led to a decrement of -0.49 and -0.29 in mean Life Years and QALYs, respectively, whilst in progression. There were similar corresponding decrements whilst in progression with the partitioned survival approach of -0.53 and -0.32.

Another contribution to the differences in overall benefit was the mean Life Years and QALYs spent progression-free. The largest benefit was found with the manufacturer’s Markov decision-analytic modeling and the partitioned survival approach, which used the same Weibull distribution to model progression-free survival. Mean Life Years (QALYs) gained of 1.18 (0.95) were found whilst progression-free. The corresponding results were more modest with the multi-state modeling at 0.81 (0.65).

The manufacturer’s Markov decision-analytic model had the largest overall benefit as a result of the largest increment whilst progression-free and the smallest decrement whilst in progression. In contrast, the smallest overall benefit was found with the multi-state modeling due to the combination of the smallest increment whilst progression-free and a relatively large decrement whilst in progression.

In Appendix 2, the results are shown separately for the observed period of the trial and the unobserved extrapolation period. The approaches were generally comparable over the observed period. However, in the unobserved extrapolation period, more of a discrepancy was apparent.

The mean costs were then incorporated to allow cost-effectiveness to be evaluated (Table 3). Details of the assumptions made with regards to mean costs as part of a full cost-effectiveness analysis can be found elsewhere.^10^

**Table 3:** Incremental Cost-effectiveness Ratios

	Partitioned Survival			Markov Decision-Analytic Modeling			Multi-state Modeling		
	RFC	FC	Incremental	RFC	FC	Incremental	RFC	FC	Incremental
Mean Life Years	5.96	5.31	0.65	5.73	4.65	1.07	5.29	4.97	0.32
Mean QALYs	4.40	3.77	0.63	4.26	3.38	0.88	3.84	3.49	0.35
Mean Total Cost	£25,369	£15,123	£10,246	£25,595	£13,978	£11,617	£25,261	£14,960	£10,301
**Cost per Life Year Gained**			**£15,694**			**£10,825**			**£31,970**
**Cost per QALY gained**			**£16,308**			**£13,189**			**£29,022**

Table 3 shows that the partitioned survival approach and manufacturer’s Markov decision-analytic modeling deemed the treatment cost-effective, with Cost per QALY gains of £16,308 and £13,189, respectively. However, the results were less conclusive when the multi-state modeling approach was used, with a Cost per QALY gain of £29,022, close to the maximum of the range of £20,000 to £30,000 WTP threshold in the UK.

## Discussion

This article has compared survival estimates of Life Years and QALYs using the multi-state modeling approach and the partitioned survival and Markov decision-analytic modeling approaches commonly used in cost-effectiveness analysis. Cost-effectiveness results were also compared between the approaches. The different assumptions used for the modeling of the transitions led to different results. In particular, the discrepancy in the results led to differing ICERs, which could affect the conclusions for cost-effectiveness. We recommend that analysts liaise with clinicians and use registry data and/or external sources to help gather evidence to ensure assumptions are realistic. In our comparison, for ease of demonstration across approaches, we used the same 15-year time horizon as in the manufacturer’s existing Markov decision-analytic model. However, when analysts are undertaking their own modeling, we advocate checking that the time horizon and extrapolation to that point is realistic in a clinical sense, and to rigorously check all other assumptions used in models. In our related analysis, where the approaches used the same assumptions (Appendix 1), it was apparent that the actual approach used had less of an effect on any discrepancy in the results than the differing assumptions made within each approach.

Multi-state modeling is an alternative, elegant way of estimating transition probabilities. This multi-state modeling approach uses the individual patient data directly to model the transitions and negates deciding on transition probabilities *a priori*. It uses the exact times of transition and, as such, does not require modeling over (arbitrary) discrete cycles, nor does it require the use of tunnel states. Additionally, the multi-state modeling approach demonstrated in this paper can incorporate parametric distributions for hazards, which, as well as allowing extrapolation of survival, can permit hazards that vary over time, if required. Given the modeling at the individual patient level, multi-state modeling also provides an alternative to microsimulation.^29^

In this illustration, we had the individual patient level data (IPD). This meant we were able to build a state-arrival extended model to help decide whether the Markov assumption was reasonable. We found evidence to suggest a violation of the assumption and proceeded to use a semi-Markov approach to relax the assumption. When modelers have insufficient power to detect violations of the Markov assumption, and have doubts over whether it holds, then a semi-Markov, multi-state modeling approach would be worth considering.

When IPD are not available, then a semi-Markov approach can still be used, if Kaplan-Meier survival curves related to all relevant transitions are available. However, if a Markov and/or state-arrival extended approach is desired without IPD, then information on relevant model coefficients will be required from an external source. The cumulative hazards necessary for the calculation of transition probabilities can be derived from this model output, instead of deriving them from direct regression modeling of the data. Functions to perform multi-state modeling without IPD are included in our prior tutorial paper.^10^

The modeling of the progression to death transition resulted in a hazard ratio for treatment of 1.408 with the multi-state modeling. It could be argued that a hazard ratio of this magnitude is over-fitting the data, given the size of the sample and the *P* value associated with the hazard ratio. However, we would argue that this should be used as a best estimate of effect for a subsequent economic evaluation, rather than just assuming no effect, which was likely due to a lack of statistical power, and that the uncertainty in model parameters be captured in sensitivity analyses that are carried out later in the evaluation. A fuller assessment of the uncertainty in the parameters is included in a tutorial-based paper, which demonstrates how to do an economic evaluation wholly in R, including deterministic and probabilistic sensitivity analyses.^10^ However, if over-fitting is of concern, shrinkage^30^ or a Bayesian approach^31^ can provide elegant solutions. We also strongly encourage modelers to consider modeling uncertainty by calculating bootstrapped confidence intervals for the transition probabilities in each model considered. This was outside the scope of this paper and has been “left on the table”.

To allow for comparisons with the Markov decision-analytic modeling adopted by the manufacturer, this illustration has been limited to a very simple model using treatment as the only covariate in the modeling of the three transitions. When IPD is available, we recommend making full use of the data and considering a multi-state modeling approach. Incorporating other covariate information—whichever modelling approach is adopted—would also be worthwhile, provided the number of patients experiencing transitions was of sufficient size, and should lead to improved predictions of transition probabilities with reduced uncertainty.

When extrapolation is required for the partitioned survival approach, deciding which method to use is not trivial. However, the extrapolation of survival can be even more complex with a state-transition approach, such as the commonly used Markov decision-analytic modeling or this multi-state modeling framework. With the partitioned survival approach, there is only one Kaplan-Meier curve at a time to consider and extrapolate for each survival outcome. With the modeling of transitions, the probabilities are not based on one outcome but on transitions that are interlinked. The models often include intermediate states with probabilities that need to represent that patients can flow in, flow out or remain in that state at any given time. When evaluating different parametric distributions, they should be considered for each transition simultaneously and this is not necessarily trivial. For example, for this illustration, six standard distributions were considered for each of the three transitions, which comes to 6^3^ = 216 combinations for each treatment before deciding on the final model. Consequently, we recommend visually assessing the fits to immediately rule out those that do not fit well from any further consideration.

In the illustration in this paper, it was assumed that transition times were known exactly for each patient and were continuous, rather than being measured discretely over a series of cycles, as in the spreadsheets often used in health economics. Multi-state modeling using the continuous-time framework is normally built with statistical software. This means that there are no spreadsheets that need to be set up ready to be populated, and the syntax-based approach means that there is a written record of what was done, all contained in just one file. Building the model is a lot less computer-intensive, and there can be considerable time savings. For example, obtaining the state occupancy probabilities from a Markov model using this framework only requires one line of code that produces output in 1 second. Even the simulation involved to produce the state occupancy probabilities from the semi-Markov model in this illustration only took 90 seconds. It is also possible to undertake multi-state modeling when times are not known exactly but are instead interval-censored using the R msm package,^32^ providing another efficient alternative to spreadsheets.

In each of the approaches presented in this paper, the assumptions used with regards to transition probabilities/hazards did not appear to be unreasonable at face value. Modelers, due to time constraints, may limit the number of approaches they consider to those with which they are most familiar. However, the comparison illustrated in this paper has highlighted that different assumptions can lead to different conclusions with regards to effectiveness and cost-effectiveness. We therefore recommend that any assumptions used are rigorously checked to ensure they are realistic. We also advise that the assumptions are subject to appropriate sensitivity analyses as part of a full cost-effectiveness analysis.

## Supplementary Material

Supplementary material
