# Supplementary material for: Estimation of Survival Probabilities for Use in Cost-effectiveness Analyses: A Comparison of a Multi-state Modeling Survival Analysis Approach with Partitioned Survival and Markov Decision-Analytic Modeling
Source: Med Decis Making. 2016 Oct 4;37(4):427–39. doi: 10.1177/0272989X16670617 (PMC5424853; doi:10.1177/0272989X16670617)
Supplement: Supplementary material [file DS_10.1177_0272989X16670617.pdf]

Appendix 1 Congruous comparison of the manufacturer's Markov decision-analytic modelling with multi-state modelling

In this appendix, a congruous comparison of the manufacturer's Markov decision-analytic modelling and the multi-state modelling approach is presented in the sense that the same assumptions were used for each approach. In order to facilitate this, the assumptions used for the transition probabilities in the manufacturer's Markov decision-analytic modelling were converted to transition hazards for use with the multi-state modelling. Transition hazards are modelled in multi-state modelling because this is more appropriate than modelling transition probabilities in a competing risks context. This is due to the one-to-one relationship between probabilities and hazards that exists when competing risks are absent no longer holding. Consequently, the calculation of the state occupancy probabilities requires combining the hazards, rather than probabilities, of all relevant transitions. However for comparison purposes, multi-state modelling based on transition probabilities was also used.

The particular assumptions made for each transition by the manufacturer in their Markov decision-analytic model were described in the "Markov decision-analytic modelling approach adopted by the manufacturer" section on page 7 of the main article. Therefore the assumptions used for each of the transitions in the multi-state modelling were similar and are described below. The assumptions for each transition are expressed in terms of cumulative hazards as this was the basis for the calculation of state occupancy probabilities in the multi-state modelling. When transition hazards were used in the multi-state modelling these were derived from the difference in cumulative hazards between two consecutive time points. When transition probabilities were used these were derived using $S(t) = \exp(-H(t))$, where S is survival, i.e. 1- the transition probability, H is the cumulative hazard and t is time. The only exception to this was for progression-free → progression which is detailed below.

- progression → death

  for each treatment arm, an exponential distribution with a hazard rate of 1/24.1791 and corresponding cumulative hazard $H(t)=1/24.1791 \times t$

- progression-free → death
  the cumulative hazard was based on the maximum of the observed rate of death whilst progression-free and an age-specific background mortality rate. The observed rates of death whilst progression-free were based on monthly probabilities of 0.0012 and 0.0039 for the RFC and FC arms respectively
- progression-free → progression
  the cumulative hazard was based on the Weibull cumulative hazard of progression or death (the compliment of staying in the progression-free state) minus the exponential cumulative hazard for progression-free -> death. Since this cumulative hazard was not based on a known standard distribution it was not possible to convert it to a transition probability in a standard way. Therefore, for the purpose of using the transition probability as input in the multi-state modelling, a similar approach to that used by the manufacturer in their Markov decision-analytic model was used for the calculation of the transition probability. This involved using the probabilities of staying in the progression-free state and that for the progression-free → death transition. The probability of staying in the progression-free state was based on the same Weibull fit to the progression-free survival data as used by the manufacturer.

Table A1 shows the incremental mean Life Years in each of the relevant health states using each of the approaches. Two methods of calculating the mean Life Years are shown. Firstly, the trapezoidal rule is used to calculate the area under the curve, the approach used in the main paper for the multi-state modelling. Secondly, the probabilities at each time point were summed together, the approach used by the manufacturer in their Markov decision-analytic modelling. The time points (measured in years) were at 1/12 increments equivalent to the monthly cycles used in the Markov decision-analytic-analytic model. The half-cycle correction used in the Markov decision-analytic-analytic model involved taking the average of the probabilities at consecutive time points. The actual results presented by the manufacturer are shown in bold.

Table A1 Incremental mean Life Years in each health state: equivalent assumptions

	mean Life Years calculated using:					
	area under the curve with trapezoidal rule			sum of transition probabilities over time		
	RFC	FC	Incremental	RFC	FC	Incremental
Mean Life Years Progression-free						
Markov decision-analytic modelling with a half-cycle correction	4.07	2.88	1.18	**4.11**	**2.93**	**1.19**
Markov decision-analytic modelling without a half-cycle correction	4.10	2.92	1.18	4.15	2.96	1.18
multi-state modelling using transition hazards	4.07	2.89	1.18	4.11	2.93	1.18
multi-state modelling using transition probabilities	4.11	2.87	1.24	4.15	2.92	1.24
Mean Life Years in Progression						
Markov decision-analytic modelling with a half-cycle correction	1.62	1.73	-0.11	**1.62**	**1.73**	**-0.11**
Markov decision-analytic modelling without a half-cycle correction	1.61	1.73	-0.11	1.62	1.73	-0.11
multi-state modelling using transition hazards	1.45	1.58	-0.13	1.45	1.58	-0.13
multi-state modelling using transition probabilities	1.58	1.73	-0.15	1.59	1.73	-0.15

It can be seen in Table A1 that the results for mean Life Years in Progression were very similar. It can be seen that the results for the individual treatments from the multi-state modelling with transition probabilities were closer to that for the Markov decision-analytic modelling than the corresponding results using transition hazards. However, in terms of incremental results, the transition hazards approach produced means that were nearer the Markov decision-analytic modelling results. This was because, whilst there was more discrepancy between the individual treatment means, the differences were comparable for each treatment resulting in a similar incremental effect.

Table A1 shows there was more of a discrepancy in results for mean Life Years Progression-free, although the results were still very similar. Regardless of the method used to calculate the means, the multi-state modelling approach using

transition probabilities produced incremental results which were higher than any of the (similar) corresponding results from the other approaches. It can be seen that the transition hazards approach was most comparable to the actual Markov decision-analytic modelling when the summing of probabilities method was used. When the trapezoidal rule was used, the results using the multi-state modelling with transition hazards and the Markov decision-analytic modelling were similar. However it was the Markov decision-analytic modelling without the half-cycle correction that most represented the actual Markov decision-analytic modelling when the trapezoidal rule was used. This suggests that using the trapezoidal rule for calculating the means (based on probabilities without a half-cycle correction) is equivalent to the summing of probabilities for which there was a half-cycle correction.

For the Markov decision-analytic modelling in the main paper, which uses a half-cycle correction, the means are calculated using the sum of the probabilities. For all other approaches no half-cycle correction is involved and the means are calculated using the trapezoidal rule. Therefore the calculation of means used in the comparison presented in the main paper would appear to be reasonable.

Appendix 2 Mean Life Years and QALYs: trial observation period of 0-4 years and extrapolation period of 4-15 years

In Tables A2.1 and A2.2 the mean Life Year and QALY results are shown separately for the observed period of the trial and the unobserved extrapolation period respectively.

Table A2.1 Mean Life Years and QALYs: trial observation period of 0-4 years

	Partitioned Survival			Markov decision-analytic model			Multi-state modelling		
	RFC	FC	Incremental	RFC	FC	Incremental	RFC	FC	Incremental
Mean Life Years	3.42	3.32	0.10	3.42	3.27	0.16	3.40	3.28	0.12
Mean Life Years Progression-free	2.85	2.42	0.43	2.85	2.44	0.41	2.87	2.37	0.49
Mean Life Years in Progression	0.57	0.90	-0.33	0.57	0.82	-0.25	0.54	0.91	-0.37
Mean QALYs	2.62	2.48	0.15	2.62	2.45	0.18	2.61	2.44	0.17
Mean QALYs Progression-free	2.28	1.94	0.34	2.28	1.95	0.33	2.29	1.90	0.39
Mean QALYs in Progression	0.34	0.54	-0.20	0.34	0.49	-0.15	0.32	0.55	-0.22

Table A2.2 Mean Life Years and QALYs: extrapolation over 4-15 years

	Partitioned Survival			Markov decision-analytic model			Multi-state modelling		
	RFC	FC	Incremental	RFC	FC	Incremental	RFC	FC	Incremental
Mean Life Years	2.54	1.99	0.55	2.30	1.39	0.92	1.89	1.68	0.20
Mean Life Years Progression-free	1.26	0.50	0.75	1.26	0.48	0.78	0.49	0.17	0.32
Mean Life Years in Progression	1.29	1.49	-0.20	1.05	0.90	0.14	1.40	1.51	-0.11
Mean QALYs	1.78	1.29	0.48	1.63	0.93	0.71	1.23	1.05	0.19
Mean QALYs Progression-free	1.00	0.40	0.60	1.01	0.39	0.62	0.39	0.14	0.25
Mean QALYs in Progression	0.77	0.89	-0.12	0.63	0.54	0.08	0.84	0.91	-0.07

It can be seen in Table A2.1 that the approaches were reasonably comparable over the observed period of the trial. However in the unobserved extrapolation period

(Table A2.2) there was an increment in mean Life Years/ QALYs gained whilst in progression with the manufacturer's Markov decision-analytic modelling. This was contrary to the rest of the approaches which found decrements.
